# Sensory evaluation of biscuits enriched with artichoke fiber‐rich powders *(Cynara scolymus* L.)

**DOI:** 10.1002/fsn3.541

**Published:** 2017-11-20

**Authors:** Francisco J. San José, Montserrat Collado‐Fernández, Rafael López

**Affiliations:** ^1^ Centro de Innovación y Tecnología Alimentaria de La Rioja CTIC‐CITA La Rioja La Rioja Spain; ^2^ Departamento de Biotecnología y Ciencia de los Alimentos Universidad de Burgos Burgos Spain

**Keywords:** By‐product, functional ingredient, revaluation, trained panel

## Abstract

The artichoke by‐products from the canning industry are mainly used for silage, being minimally revaluated. The ways of extraction of by‐products of artichoke into fiber‐rich powders modify their industrial applications in biscuits, as the sensory evaluation may change compared with the reference fiber (Pea fiber, P) used with commercial biscuit. In this sensory study biscuits enriched with fiber‐rich powders of artichoke (W, Ca) are compared with biscuits with the same percentage of the reference fiber (P) and control biscuits without fiber (B). For most of the sensory attributes of the biscuits enriched with artichoke fiber‐rich powders were perceived similar to the biscuits with the commercial reference fiber (P). The good sensory behavior of the biscuits with artichoke fiber‐rich powders during two storage conditions applied may confirm that the artichoke by‐products would be a suitable substitute for pea fiber in oven‐baked products, like wholemeal biscuits with high‐fiber content.

## 1 INTRODUCTION

Wholemeal biscuits with a high‐fiber content constitute one of the rising sectors of the food industry in recent years, because of their possibilities of formulation and relatively low production costs. Consumer trends demand more natural products with better nutritional characteristics which can complement nutritional deficiencies, like the low fiber ingestion (Arrillaga & Martínez, 2006; Milte, Thorpe, Crawford, Ball, & McNaughton, 2015; Mongeau, Brassard, & Verdier, 1989).

In this respect, the simple incorporation of dietary fiber in the formulation of biscuits is an easy method to increase the functionality of the biscuits, with minimal costs (Gallagher, O'Brien, Scannell, & Arendt, 2003; Smith, 2011). Numerous studies confirm that vegetable fiber prevents diseases such as gastrointestinal disorders, duodenal ulcer, constipation, hemorrhoids, Type II diabetes, obesity, cardiovascular diseases, (Afaghi, Kordi, & Sabzmakan, 2015; Vitaglione, Napolitano, & Fogliano, 2008) and kidney stones (Sorensen et al., 2014).

The dietary fiber composition of the artichoke is high; singling out its high composition of inulin, which can be considered its most important industrial and functional composite and a prebiotic compound itself (Muzzarelli et al., 2012).

From an environmental perspective, the revaluation of vegetable by‐products is also critical, since it increases the economic viability of waste minimization processes. Usually, those by‐products (70% by weight are by‐products of artichoke) are only used as animal feed, being minimum their economic revaluation. Assuming that the by‐products have a composition similar to the edible part of the artichokes, these by‐products can be a promising source of new value‐added compounds such as phytochemicals and fiber (Ruiz‐Cano et al., 2014). Note that recent publications of revaluation indicate that vegetable by‐products of the canning industry (asparagus) (Fuentes‐Alventosa et al., 2009; Nindo, Sun, Wang, Tang, & Powers, 2003), chard, cardoon, green beans, etc. (Randhawa, Khan, Javed, & Sajid, 2015) have good functional qualities, such as Water or Oil holding capacity, and high content of antioxidant compounds (polyphenols, etc.) and fiber (soluble and insoluble). All those functional qualities are really demanded by manufactures of enriched products (such as biscuits, meat, dairy products) who eventually could select vegetal fiber‐rich powder over other fibers extracted from grains (Rodríguez, Jiménez, Fernández‐Bolaños, Guillén, & Heredia, 2006), rice (Gul, Yousuf, Singh, Singh, & Wani, 2015), tubers or roots as Fibrex^®^ (sugar beet), Raftilose^®^ (chicory) with less functional qualities.

However, there are no commercial products of food fiber‐rich powders of artichoke yet, although it would be very interesting to study their possible industrial appliance. Thus, the aim of this study was to describe the biscuits enriched with artichoke fiber‐rich powders, compared to biscuits formulated with commercial reference fiber (pea) and control biscuits without fiber. The shelf‐life study of the biscuits was carried out by investigating the evolution of the sensorial qualities during storage at ambient (25°C) and accelerated (45°C) temperature.

## 2 MATERIAL AND METHODS

### 2.1 Ingredients and preparation of the biscuits

The biscuits were formulated in triplicate following the recipe of Whitley (1970) with slight modifications in the percentages of the ingredients since 4% (w/w) of plant fiber was added. The choice of this percentage of fiber was determined by an optimization study from which it was concluded that with 4% of plant fiber of both varieties (commercial reference fiber P and fiber‐rich powders from artichoke Ca and W) obtained the maximum sensorial acceptability (data not shown). The commercial pea fiber (P) was obtained on the Spanish market of food additives and the two fiber‐rich powders were produced in our laboratory by liquid extraction of artichoke by‐products. Two different extraction liquids were used: distilled water (W) and a solution of 1% (w/w) of CaCl_2_^.^5H_2_O (pH 6.5) (Ca). In the control biscuits, the 4% (w/w) of fiber was replaced by wheat flour.

**Figure 1:**
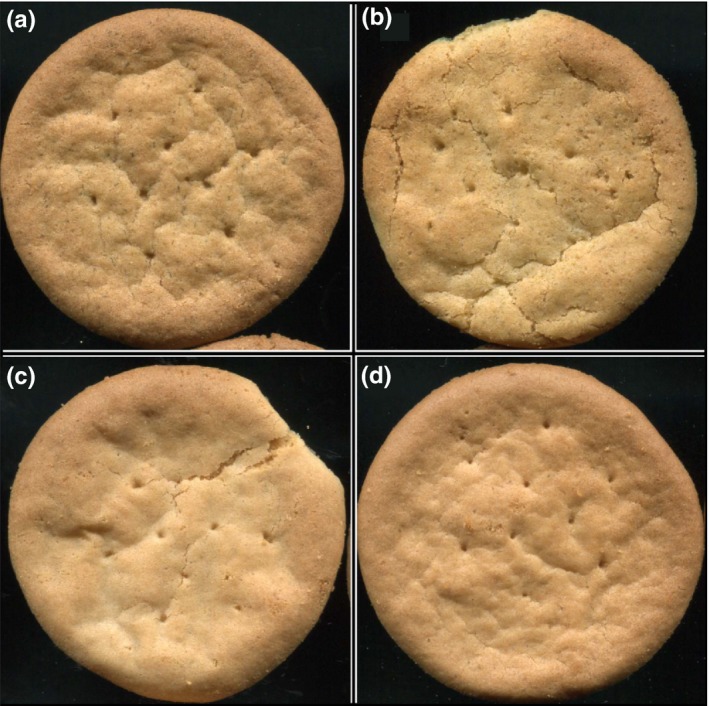
Biscuits after oven‐baking. (a) Biscuit W, (b) Biscuit Ca, (c) Biscuit B, (d) Biscuit P. Biscuit codification (B [without fiber], P [pea], W [water], Ca [1% CaCl_2_.5H_2_0])

The dough was kneaded with a pilot mixer during 10 min at minimum speed. Subsequently, the dough was laminated to a thickness of 5 mm, using a manual sheet pasta machine. The round form of the biscuits was obtained manually, using a cutting cylinder (die) of 60 mm in diameter. After making eight evaporation holes in the dough with a fork, the biscuits were baked in an electrical convection oven at a temperature of 180 ± 10°C during 17 min (Figure 1).

Afterwards, the biscuits were cooled at ambient temperature (18–20°C) and wrapped in heat‐sealed bags of high‐density polyethylene. The biscuits were divided into two groups as a function of the storage conditions. As the control for each group of biscuits, the values were taken at time 0, which was the day after preparation.

### 2.2 Storage

The wrapped biscuits were stored in two incubation chambers (Incudigit Selecta Spain), where temperature and humidity were kept constant.

One of the chambers simulated storage at room temperature (25°C, RH 55%), while the other simulated conditions of accelerated storage (45°C, RH 55%) (Yang et al., 2013), forcing the appearance of defects in the biscuits by the increase in temperature.

The biscuits stored under ambient conditions were analyzed bimonthly, during the 4 months, while the biscuits stored under accelerated conditions were analyzed every 3 months until completion of the 6 months of storage. By comparing the results of both storage procedures, it intended to conclude at which time under ambient conditions the defects were detected, generated under accelerated storage.

### 2.3 Sensory analysis

The sensory evaluation was carried out in the tasting room of CITA‐CTIC La Rioja. The sensory panel consisted of nine judges trained in the use of the sensory profiles method (Lawless & Heymann, 2010). The panelists were trained with commercial biscuits and prototypes prepared in pilot plants, since the biscuits formulated with fiber‐rich powder were also prepared in a pilot plant. By means of this training, a specific terminology for the sensory characteristics and ranges for each attribute was agreed upon. All the trained judges of the internal sensory panel passed the basic taste test, the odor test and the color vision test, and their evaluation capacity was routinely verified by way of individual control cards. The vocabularies of the sensory attributes were agreed upon in order to describe the differences between samples. The intensity of the attributes was classified on non‐structured, continuous graphic scales. The scales were 10 cm in length and it was verbally agreed that the end on the left‐hand side of the scale corresponded to the lowest intensity (value 0) and the end on the right‐hand side to the highest intensity of the attribute (value 10); while the intermediate point was the value of the commercial standard biscuits (value 5).

The freshness of the samples was evaluated by the presence or absence of characteristic flavor/taste, color, texture, appearance, overall acceptance. For the biscuit samples without accelerated storage, a fresh sample should have a taste and flavor similar to the standard. Therefore, the absence of characteristic flavor and taste was rated below the standard value, while flavor and taste were rated above the standard value, up to very roasted or burnt as the upper end of the scale.

Color defined the degree of toasting and the outer ends of color went from bread crust of white tin loaf to toasted wholemeal bread. Hardness was expressed as the resistance of the biscuit to breaking upon pressure of the front teeth during biting. For a hard biscuit, the strength needed for breaking would increase (the standard outer ends of texture went from the hardness of toasted bread rusks to that of wholemeal “grissini”‐type rusks of 0.5 cm in diameter).

Appearance is related to the geometry and the structure of the biscuits, and the outer ends of appearance went from an irregular biscuit with an irregular surface produced by evaporation bubbles, to a very compact biscuit with a smooth surface.

**Figure 2:**
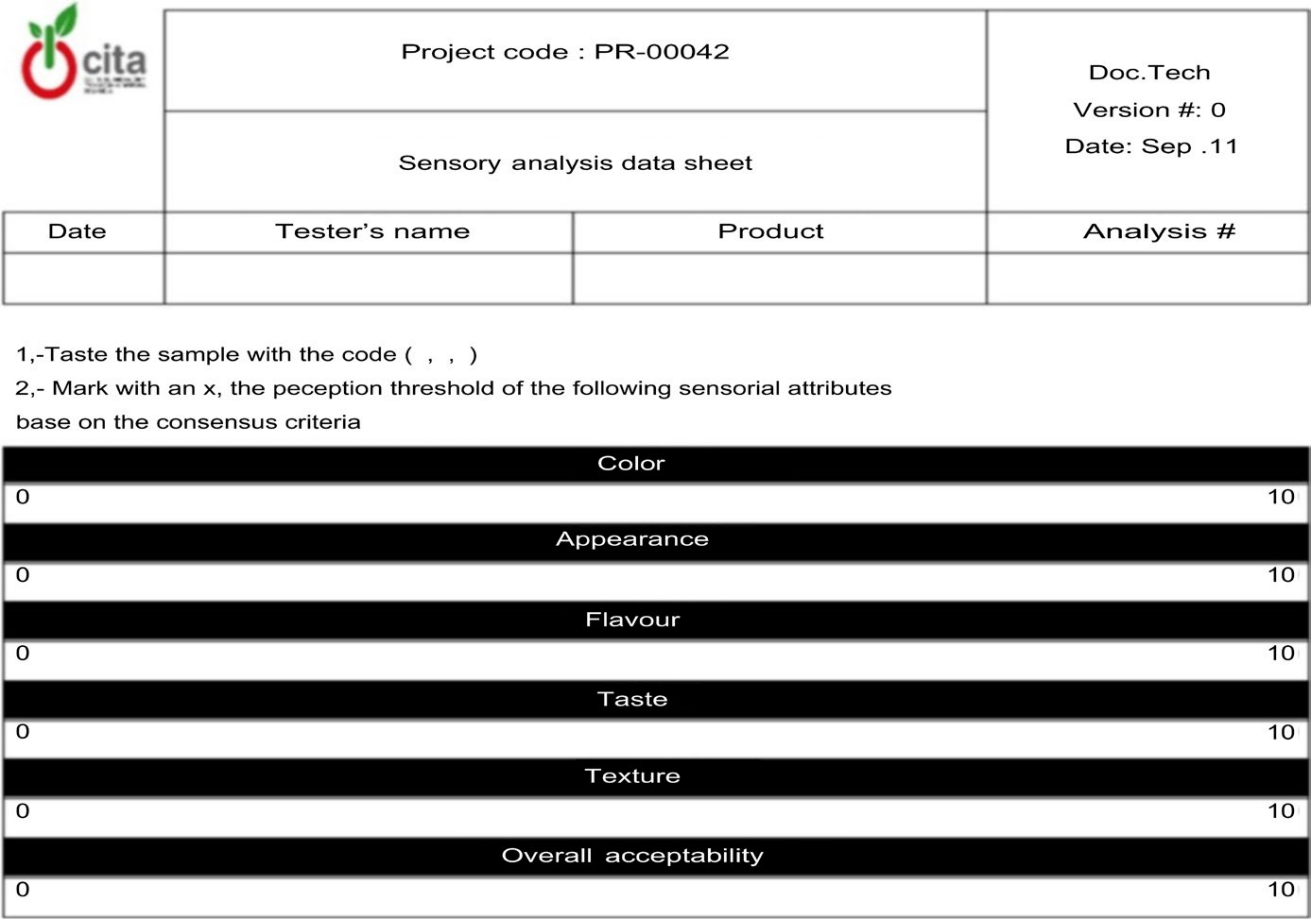
Tasting ballot

Overall acceptability is a mixture of all of the sensory attributes and therefore the outer ends of the scale go from: very little like the commercial standard biscuit, with a very light color, little flavor and taste of biscuit, a very crumbly and soft biscuit, to a very dark biscuit, with a strong taste of toasted, a very hard and compact biscuit, at the upper end. In order to be able to describe the possible sensory defects, like the appearance of strange flavors and odors caused by the accelerated storage, the consensus on the sensory variables taste and flavor was differentiated. For the attributes characteristic flavor and taste, the freshness of the sample was rated as the point in the middle of the non‐structured scale. Thus, the absence of characteristic flavor and taste was rated below the standard value, like loss of characteristic flavor or taste when tastes or smells related to oxidative processes were detected, being a strong flavor and taste of rancidity the lower end of the scale. This nuance in the description of the sensory outer ends of flavor and taste caused the redefinition of the overall acceptability of the biscuits, in the lower end of the scale of overall acceptability, where the biscuits have a very light color, a strong taste and smell of rancidity, and are very soft and crumbly (Figure 2).

The presentation of the biscuit samples was performed in a blind fashion, coding each sample with a random numeric code of three digits. The order of presentation of the biscuits was balanced using all of the presentation combinations possible on the tasting sheet. The characterization of the products was carried out under daylight lighting and in portable cabins within the sensory laboratory. The ration of consumption was one whole biscuit of each reference, maintained at a temperature of 25°C. Water was served to the evaluators for cleaning of the mouth between the different samples. The samples were judged without replicate. With the aim of testing the reliability of the results, the control biscuit was introduced two times in the evaluations, randomly between other samples.

### 2.4 Sensory samples

The samples tested during the sensory analysis were samples randomly selected from each of the three pilot plant batches. The three session of each replica of samples were performed with a time lapse of 2 days. Before the sensory analysis sessions, the samples were tempered for 30 min, at a temperature of the sensory analysis room (25°C). The presentation of the sample to the panelist was as follows: using white light, the four hold biscuits were presented together on a piece of white paper divided into four equal parts, in which the codes of the biscuit were printed randomly. The panelists were instructed to taste the samples clockwise starting from the upright quarter.

### 2.5 Statistical analysis

Statistical analysis of the sensorial measurements was carried out with the sensory analysis software Panel Check. The data were expressed as Mean ± Coefficient of variation (CV). Two‐way ANOVA (1 rep) sample means and LSD, and principal component analysis (PCA‐statis) were applied. The PCA decomposes the viability of a group of data in principal components and tracks the panelist in the principal component space according to their valuations of the samples together with the average classifications of the samples or attributes.

## 3 RESULTS AND DISCUSSION

### 3.1 Shelf‐life test at room temperature

**Figure 3:**
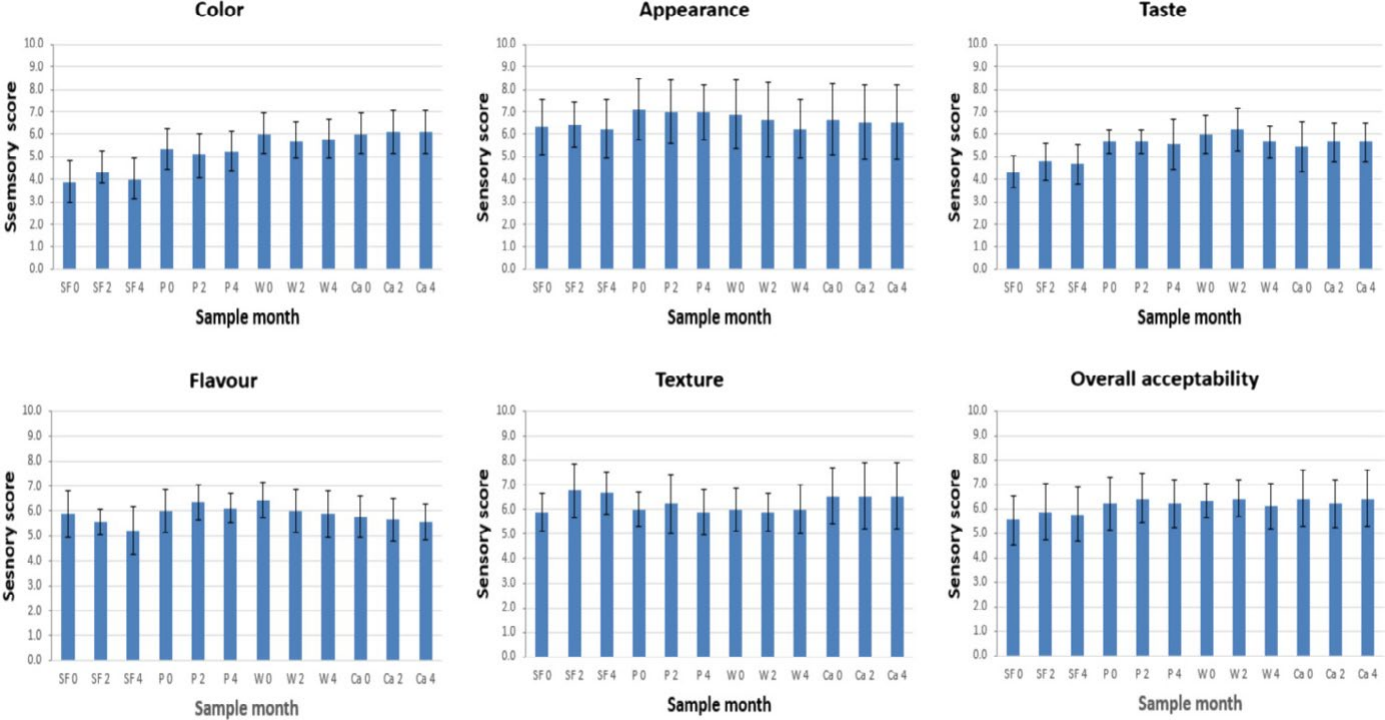
Organoleptic properties (Color; Appearance, Taste, Flavor, Texture, Overall acceptability) of biscuits formulated without fiber (B), and with fiber of Pea (P) and fiber‐rich powder of Artichoke (W, Ca), storage at room temperature (0, 2 and 4 months). Values are means ± *SD* (*n* = 9)

**Figure 4:**
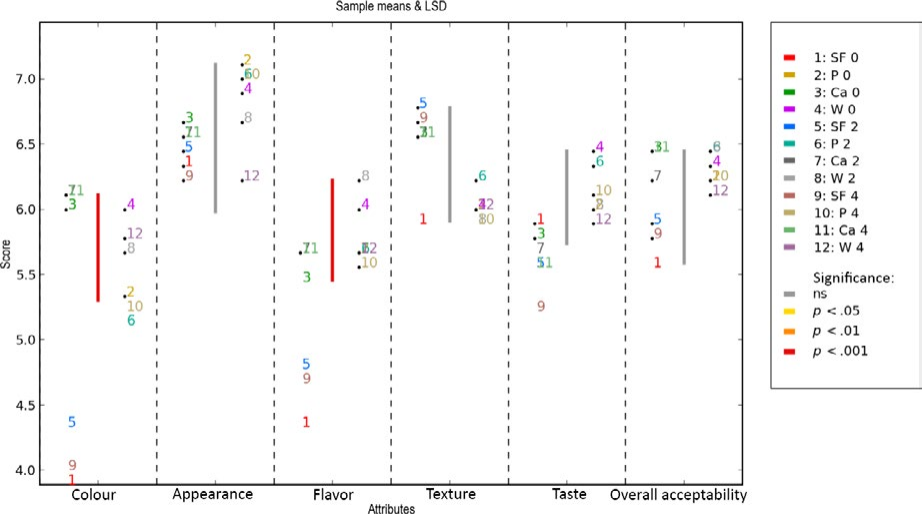
Two‐way ANOVA (1 rep) sample means & LSD. Storage of biscuits formulated without fiber (B), and with fiber of Pea (P) and fiber‐rich powder of Artichoke (W, Ca), at room temperature and with accelerated storage for (0, 2, 4) months Values are means ± *SD* (*n* = 9)

As can be observed in Figures 3 and 4, color and taste were the two sensory qualities for which all the groups of biscuits were significantly different (*p* < .001). With respect to color, as was expected, the biscuits without fiber (B) had a lighter color than the rest, followed by the pea biscuits (P), while the biscuits formulated with artichoke fiber‐rich powders (W, Ca) were significantly darker, mainly due to the darker color of the artichoke fiber‐rich powders (Figure 1).

Regarding the variable taste, it was observed that all the biscuits enriched with fiber had a more intense taste than the biscuits without fiber (B). When comparing the biscuits with artichoke fiber‐rich powders (W, Ca) with the biscuits formulated with pea fiber, it was observed that their taste was similar.

With respect to the overall acceptability, biscuits B were more similar to the standard biscuit, because of their more neutral taste and color. Similar results were found by other authors for wholemeal wheat biscuits in which part of the flour was replaced by another functional ingredient (Akubor & Badifu, 2004; Cheng & Bhat, 2016; Kohajdová, Karovičová, & Magala, 2013). The panelists preferred the lighter color of the wholemeal wheat biscuits when a high level of replacement of wheat flour caused a low acceptance by the panelists with respect to the attributes of color. In our case, the acceptability of the biscuits formulated with artichoke fiber‐rich powders was similar to that of the biscuits with pea fiber. It can also be observed, from the texture, that the biscuits with fiber were significantly harder than the biscuits without fiber. For the rest of the sensory qualities (appearance and flavor), all groups of biscuits were similar and their values remained constant during storage at ambient temperature.

**Figure 5:**
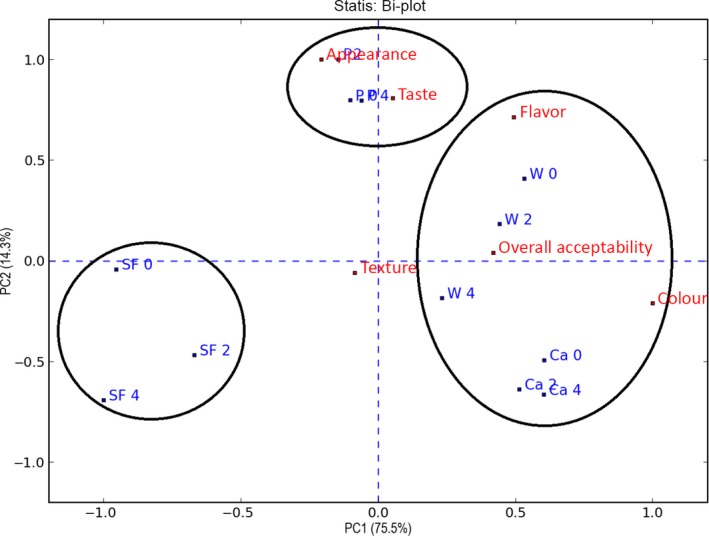
PCA‐statis plot of the sensory profile of the biscuits by descriptive method. Storage of biscuits formulated without fiber (B), and with fiber of Pea (P) and fiber‐rich powder of Artichoke (W, Ca), at room temperature and with accelerated storage for (0, 2, 4) months. Values are means ± *SD* (*n* = 9)

PCA‐statistical analysis explained 89.8% of the variation in the sensory attributes of the biscuits; principal component 1 (PC1) explained 75.5% and the second component (PC2) 14.3% (Figure 5). In the first principal component, the variable color and overall acceptability were correlated with the biscuits formulated with artichoke fiber‐rich powders (Ca, W) separating them from biscuits B and P. Thus, if the color of the biscuits is darker, this is related to an increase in the values of overall acceptability.

Regarding the second principal component, taste is the sensory variable that produced the major differentiation of the groups, separating them in biscuits with pea fiber, biscuits with artichoke fiber‐rich powders and biscuits without fiber.

When looking at the groups that were formed in the PCA‐statis plot individually, it is observed that throughout the 4 months of storage at ambient temperature the groups of biscuits formulated with vegetal fibers remained grouped more closely. This is explained by a lack of significant variation in the sensory qualities and therefore, due to an effect of protection of the sensory qualities by the enrichment of the biscuits with vegetal fiber, or at least an effect of masking the changes in color and taste.

### 3.2 Accelerated shelf‐life test

**Figure 6:**
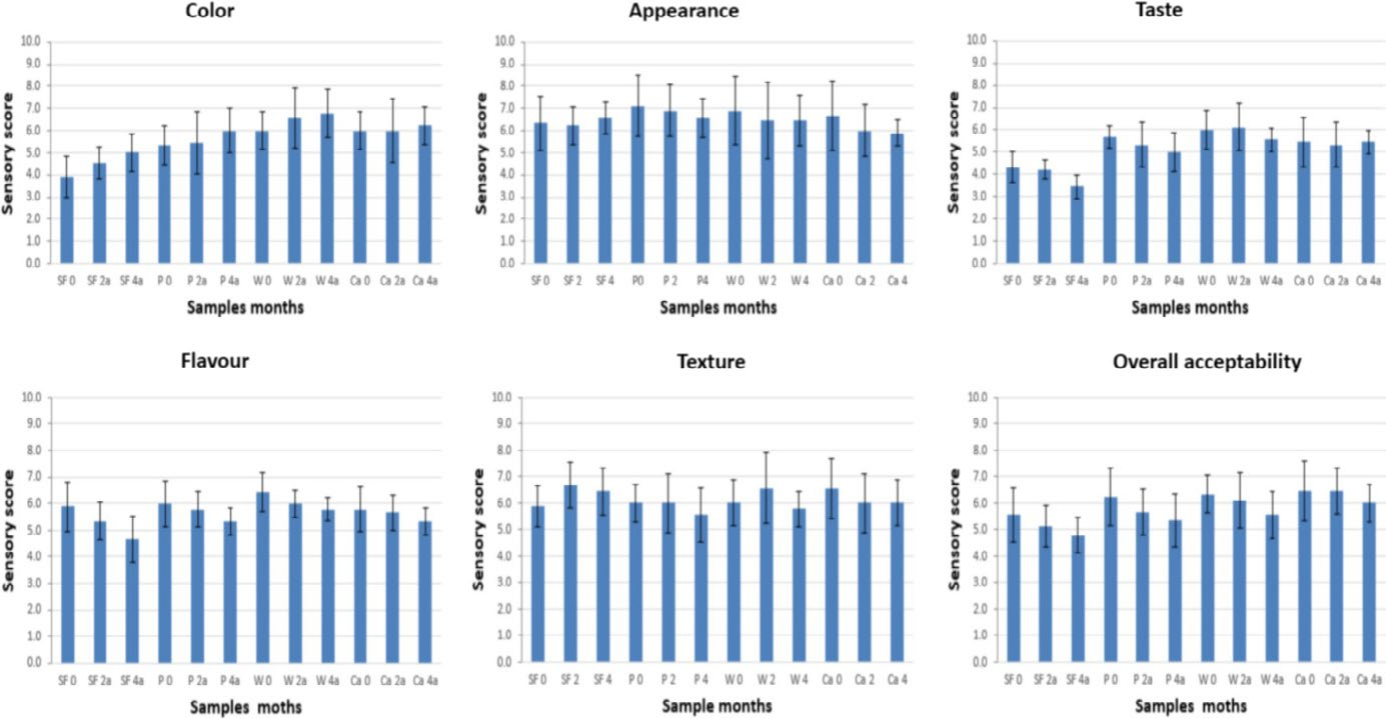
Organoleptic properties (Color; Appearance, Flavor, Taste, Texture, Overall acceptability). Storage at accelerated temperature of biscuits formulated without fiber (B), and with fiber of Pea (P) and fiber‐rich powder of Artichoke (W, Ca), for 0, 2, 4 months. Values are means ± *SD* (*n* = 9)

**Figure 7:**
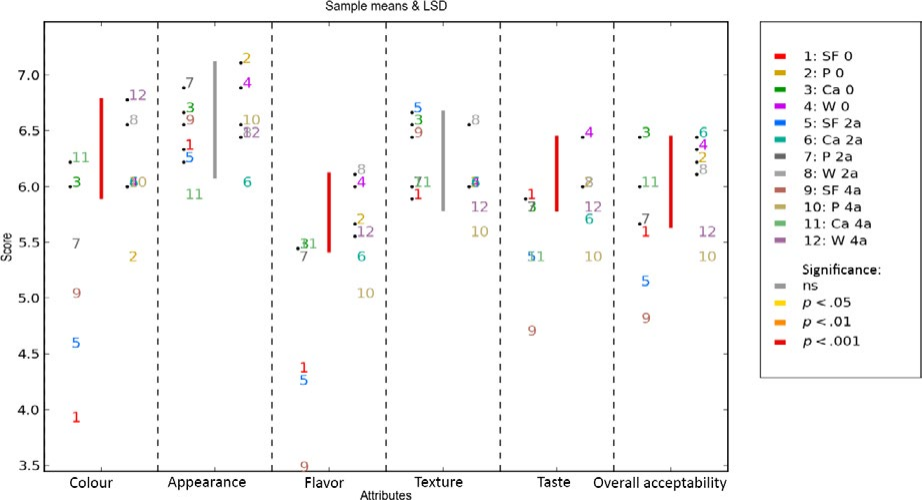
Two‐way ANOVA (1rep) sample means & LSD. Storage at accelerated temperature of biscuits formulated without fiber (B), and with fiber of Pea (P) and fiber‐rich powder of Artichoke (W, Ca), for 0, 2, 4 months. Values are means ± *SD* (*n* = 9)

As can be observed in Figures 6 and 7, during accelerated storage the score of the biscuits without fiber (B) significantly decreases for the sensory variables taste and flavor, diminishing their overall acceptability. This change in sensory perception is caused by the formation of aromatic compounds, of unpleasant taste and flavor, by‐products of lipid oxidation (Talbot, 2010) like 2,4‐decadienal y 2,4‐ heptadienal (Cheng & Bhat, 2016) and hexanal (Sakač et al., 2016).

With respect to color, only the color of biscuits B significantly increased during accelerated storage. This was partly because of the fact that the initial color of the biscuits B was lighter than that of the rest and therefore small variations in color were more perceptible by the panelist than in the biscuits with plant fiber, which were darker due to the color of the fiber.

When the sensory valuation of texture and appearance is observed during accelerated storage, no significant differences are found between the groups of biscuits. The biscuits maintained similar values throughout the storage period and both sensory variables had a constant behavior during accelerated storage. With respect to the overall acceptability, only the biscuits without fiber had a significantly lower appreciation at the end of accelerated storage.

**Figure 8:**
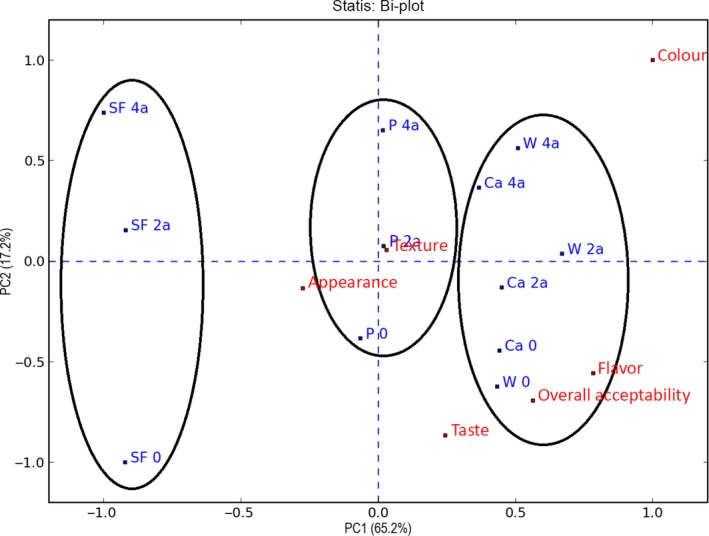
PCA‐statis biplot of the sensory profile of the biscuits by descriptive method. Storage at accelerated temperature of biscuits formulated without fiber (B), and with fiber of Pea (P) and fiber‐rich powder of Artichoke (W, Ca), for 0, 2, 4 months. Values are means ± *SD* (*n* = 9)

For the biscuits subjected to accelerated storage, the PCA‐statis analysis explained a total of 82.4% of the variation in the sensory attributes of the biscuits; the first principal component 1 (PC1) explained 65.2% and the second (PC2) 17.2% (Figure 8). This effect on the decrease in the percentage of explanation of the first principal component is caused by the conditions of accelerated storage and the increase in the explanation of the model of more sensory variables, like taste and flavor.

When comparing Figure 8 with Figure 5, it is observed that in both figures the explanation of the different biscuit groups along the first principal component was due to the color of the fiber used. Since these clear differences between biscuits without fiber and the rest were maintained throughout accelerated storage, and because the biscuits formulated with fiber‐rich powders of artichoke (W, Ca) could not be distinguished, the percentage of explanation of the first principal component decreased.

On the contrary, the second principal component increases the explanation of each group of biscuits, as a result of the darkening of the biscuits during storage, and due to that accelerated at 2 and 4 months (2a, 4a) cookies are separated vertically cookies control (0). Feel this differentiation greater in them cookies of a color clearer, as the biscuits B and P, where small changes in color are more perceptible to the panelists.

The sensory variables overall acceptability, taste and flavor were correlated with the biscuits enriched with artichoke fiber‐rich powder at the beginning of the accelerated test (Ca 0, W 0). On the contrary, the variables appearance and texture are not explained well by the model, since they are close to the centre of both principal components.

In conclusion, the evolution of the sensory qualities of the biscuits formulated with artichoke fiber‐rich powders (W, Ca) was similar to that of the biscuits formulated with pea commercial reference fiber (P), and thus that its better sensory appreciation would be maintained throughout the accelerated storage.

## 4 CONCLUSIONS

The sensory description of the biscuits with fiber showed that the variables taste and color were the sensory qualities that significantly differentiate them from the biscuits without fiber (B).

The taste of all biscuits enriched with fiber was appreciated as similar by the panelist. With respect to color, the use of both artichoke fiber‐rich powders (Ca, W) modifies the color of the biscuits, making them darker than the biscuits formulated with pea fiber, due to its more neutral color. However, initially, the overall acceptability of the biscuits formulated with both artichoke fiber‐rich powders was similar to that of the biscuits formulated with pea fiber (P).

When the biscuits were subjected to accelerate storage, significant changes in taste and color were produced in the biscuits without fiber (B), since color and taste in these biscuits are more neutral, and therefore small variations are more easily detected by the panelist. It was also demonstrated that the biscuits formulated with artichoke fiber‐rich powders (Ca, W) had a similar behavior during storage to that of the biscuits formulated with the commercial reference fiber (P).

These results conclude that the utilization of fiber‐rich powders from artichoke by‐products (Ca, W) can be a viable alternative for the enrichment of biscuits as well as other oven‐baked products and that it could be a suitable substitute for commercial pea fiber.
